# Modeling Alcohol Use Disorder Severity: An Integrative Structural Equation Modeling Approach

**DOI:** 10.3389/fpsyt.2013.00075

**Published:** 2013-07-29

**Authors:** Nathasha R. Moallem, Kelly E. Courtney, Guadalupe A. Bacio, Lara A. Ray

**Affiliations:** ^1^Department of Psychology, University of California Los Angeles, Los Angeles, CA, USA; ^2^Brain Research Institute, University of California Los Angeles, Los Angeles, CA, USA; ^3^Department of Psychiatry and Biobehavioral Sciences, University of California Los Angeles, Los Angeles, CA, USA

**Keywords:** alcohol use disorder severity, structural equation modeling, alcoholism, DSM-IV-TR symptom count, affective symptoms, motivation to change

## Abstract

**Background:** Alcohol dependence is a complex psychological disorder whose phenomenology changes as the disorder progresses. Neuroscience has provided a variety of theories and evidence for the development, maintenance, and severity of addiction; however, clinically, it has been difficult to evaluate alcohol use disorder (AUD) severity.

**Objective:** This study seeks to evaluate and validate a data-driven approach to capturing alcohol severity in a community sample.

**Method:** Participants were non-treatment seeking problem drinkers (*n* = 283). A structural equation modeling approach was used to (a) verify the latent factor structure of the indices of AUD severity; and (b) test the relationship between the AUD severity factor and measures of alcohol use, affective symptoms, and motivation to change drinking.

**Results:** The model was found to fit well, with all chosen indices of AUD severity loading significantly and positively onto the severity factor. In addition, the paths from the alcohol use, motivation, and affective factors accounted for 68% of the variance in AUD severity. Greater AUD severity was associated with greater alcohol use, increased affective symptoms, and higher motivation to change.

**Conclusion:** Unlike the categorical diagnostic criteria, the AUD severity factor is comprised of multiple quantitative dimensions of impairment observed across the progression of the disorder. The AUD severity factor was validated by testing it in relation to other outcomes such as alcohol use, affective symptoms, and motivation for change. Clinically, this approach to AUD severity can be used to inform treatment planning and ultimately to improve outcomes.

## Introduction

Alcohol dependence is a complex psychological disorder characterized by excessive and compulsive alcohol use, the development of tolerance and withdrawal, and overall functional impairment [American Psychiatric (1)]. Traditionally, alcohol abuse and dependence have been conceptualized as categorical, that is, either a patient meets diagnosis or not. However, it remains unclear whether this all-or-none model is capturing the complete picture of alcohol use disorder (AUD). Utilizing a categorical construct makes it difficult to capture the full spectrum of AUDs including the multiple problem areas that are associated with excessive drinking. Examining the full spectrum of AUD severity may elucidate some of the complexities associated with the progressive, chronic, and relapsing nature of the disorder.

Multiple neurobiological theories have mapped out the progression from alcohol use to alcohol dependence. One theory hypothesizes that addiction is the result of the shift from goal-directed actions to habits and ultimately, to compulsive drug-seeking and taking (2, 3). This transition from deliberate drug use to habitual responding has been hypothesized to reflect a shift from prefrontal cortical activation to striatal regions and from ventral to dorsal subregions (3). The neuroadaptation associated with the various stages of addiction may also affect executive control over behavior, which promotes habit-forming behavior (3). The incentive sensitization theory further proposes that neuroadaptation occurs during disorder progression in which the brain circuitry becomes hypersensitive to drugs and drug-associated cues [see review by Robinson and Berridge (4)], and this hypersensitivity appears to be persistent for years (5, 6). These theories suggest meaningful neurobiological adaptations occur across the progression from alcohol use to alcohol dependence, yet their translation to clinical samples has been limited at best.

Data to support these theories has come from both preclinical and human samples. Previous studies found that moderate/heavy drinkers reported greater stimulant-like effects after acute alcohol dose as compared to light social drinkers [e.g., (7)]. These findings suggest that heavy drinkers may be more sensitive to the rewarding and pleasant effects of alcohol, placing them at greater risk for the development of an AUD. Although neurobiological theories and more recent preclinical findings have led to a better understanding of the progression of AUD severity, these have yet to be applied clinically (8). In clinical settings, determining whether or not a patient meets criteria for AUD does little to inform the necessary treatment, as the impact on functioning and stage of progression (i.e., severity) warrant the most clinical attention. In fact, in following the current DSM-IV diagnostic criteria, some patients may only meet criteria for one or two out of the four symptoms of dependence and none of the three symptoms of abuse therefore meeting criteria as a diagnostic orphan. These patients may be experiencing significant functional impairment but fail to receive treatment due to a lack of dimensionality in diagnosis. The switch to DSM-5 will include a change in the diagnostic criteria of AUD such that the abuse and dependence symptoms of the DSM-IV will be combined. Additionally, the diagnostic criteria of “legal problems” will be replaced by the symptom “drug craving.” This move to a singular AUD in DSM-5 is accompanied specification of severity level (mild, moderate, severe) which is based on the number of symptoms endorsed. This highlights the growing awareness of the importance of clinical severity in diagnosis and treatment planning.

To date, it has been difficult to evaluate AUD severity among patients as it is challenging to simultaneously capture the multiple dimensions such as withdrawal, craving, and consequences associated with AUD. Thus, a model incorporating these dimensions would be especially useful in providing a broader conceptualization of AUD severity. To achieve this goal, the present study validates an AUD severity factor comprised of quantitative scores from self-report and interview assessments that intentionally measure the multiple dimensions of clinical impairment observed across the progression of the disorder. There have been other efforts to identify latent constructs of AUDs in large epidemiologic samples (9) and to develop comprehensive assessments of the alcohol problem continuum in young adults (10). This study complements those efforts by focusing on a community-based sample of problem drinkers, by using well-validated clinical scales that capture multiple dimensions of alcohol use problems and symptoms, and by applying a data-driven structural equation modeling (SEM) approach. Additionally, our efforts to consolidate across multiple dimensions at a clinical level match well with other studies that aim to combine clinical variation of alcohol consumption and related problems at the level of treatment outcomes (11). To that end, this study seeks to validate an AUD severity model comprised of multiple dimensions of AUD phenomenology, such as diagnostic symptoms, broad-spectrum withdrawal, craving, and psychosocial consequences and examines it in relation to relevant clinical constructs, namely alcohol use, affective symptoms, and motivation for change.

## Materials and Methods

### Participants

Non-treatment seeking problem drinkers were recruited from the greater Los Angeles area through flyers, print, and online advertisements as part of a larger-scale alcohol administration study (12). Inclusion criteria consisted of the following: between the ages of 21 and 65; self-identify as having problems with alcohol; and consuming a minimum of 48 standard drinks per month. Exclusionary criteria were: current treatment for alcohol problems, history of treatment in the 30 days before enrollment, or currently seeking treatment; current DSM-IV diagnosis of dependence on any psychoactive substances other than alcohol and nicotine; lifetime DSM-IV diagnosis of schizophrenia, bipolar disorder, or other psychotic disorder; current use of psychoactive drugs, other than marijuana, as determined by a positive urine screen; living with someone who participated in this study; and not having a drink within 21 days of the phone screening.

### Procedures

Interested individuals called in to the laboratory and completed an initial telephone screening interview in order to assess for eligibility. During this initial screening, participants were asked about their alcohol use and any diagnosis of a psychiatric disorder. Participants who reported they were interested in treatment for their AUD were excluded from the study. Treatment referrals were provided to these participants as a later phase of the protocol included an alcohol administration. Following the telephone screen, eligible participants were invited to the laboratory in the psychology department of the University of California Los Angeles for an in-person session in which they read and signed an informed consent form and completed a series of questionnaires and interviews. Participants were compensated $40 for completing the in-person session. This study was approved by the Institutional Review Board of the University of California Los Angeles.

### Measures

Demographic information was collected including age, sex, ethnicity, and education. In addition, multiple interviews and self-report measures evaluating alcohol use and problems, depression and anxiety symptomatology, and motivation for change were administered as described below. All interviews were conducted by bachelor’s level interviewees or graduate students under the training and supervision of a licensed clinical psychologist (LAR).

#### AUD severity factor

The Structured Clinical Interview for DSM-IV (SCID) (13) was used to determine eligibility based on diagnostic criteria for AUD and exclusionary psychiatric disorders. DSM-IV symptoms of alcohol abuse and alcohol dependence were recorded for a total of 11 possible symptoms (4 of abuse and 7 of dependence) comprising of the indicator variable COUNT. Alcohol withdrawal was assessed using the Clinical Institute Withdrawal Assessment – Alcohol Revised [CIWA-AR; (14)]. A total score was tabulated for each participant to comprise the indicator variable CIWA. The Penn Alcohol Craving Scale (PACS) is a 5-item, self-report measure of craving for alcohol during the previous week (15). A total PACS score was calculated for each participant and included in the model as the indicator variable PACS. A total score was also calculated from the Alcohol Dependence Scale (ADS) (16), a 25-item scale that measures alcohol dependence symptoms over the past 12 months. The ADS assesses problems that are relevant for alcohol dependent drinkers (17, 18). The Drinker Inventory of Consequences [DrInC-2R; (19)] provided a baseline description of the number and frequency of various drinking consequences. The five subscales were summed to provide a single indicator variable of negative consequences of drinking (DRINC). Thus, the five indicator variables for the AUD severity factor include (i) alcohol abuse and dependence symptom count (COUNT), (ii) alcohol dependence scale (ADS), (iii) alcohol craving score (PACS), (iv) negative drinking-related consequences (DRINC), and (v) alcohol withdrawal score (CIWA). In addition, to investigate the influence of the categorical DSM-IV diagnostic criteria for alcohol abuse and dependence, a second model was estimated with the omission of the alcohol dependence symptom count (COUNT) indicator variable. In brief, the measures used in this study are valid, reliable, and widely accessible to researchers and clinicians.

#### Alcohol use

The 30-day timeline follow-back (TLFB) interview (20) was used to assess drinking behavior. The TLFB is a calendar-assisted self-report method with high reliability and validity that was used to obtain a stable and detailed baseline of quantity and frequency of drinking, including alcohol binges. According to the National Institute on Alcohol Abuse and Alcoholism (NIAAA) guidelines, an alcohol binge was defined as consuming four or more drinks within a given episode for women and five or more drinks for men. The following indicator variables of alcohol use were derived from the 30-day TLFB: (i) average drinks per drinking day (DPDD) and (ii) percent binge drinking days (Binge%).

#### Depression and anxiety symptoms

To assess for affective symptoms, the Beck Anxiety Inventory (BAI) and the Beck Depression Inventory, Revised (BDI-II) were used. The BAI includes 21 items and surveys anxiety symptomatology including physical and cognitive indicators of anxious mood. The BDI-II is a 23-item measure of depressive symptomatology, widely used in psychological research and practice. Therefore, the indicator variables derived for the affective symptoms factor are: (i) BAI sum score (BAI), and (ii) BDI-II sum score (BDI).

#### Motivation for change

The Stages of Change Readiness and Treatment Eagerness Scale (SOCRATES) is a 19-item measure of motivational processes associated with the Transtheoretical Model. The SOCRATES has been shown to be a valid and reliable measure of readiness for change (21). The following subscales of the SOCRATES were used as indicator variables for the motivation factor: (i) recognition of alcohol-related problems (RECOG), (ii) uncertainty about drinking (AMBIV), and (iii) taking action to change drinking (STEPS).

### Data analytic plan – structural equation modeling

A SEM approach was used to (a) verify the latent factor structure of the indices of AUD severity, comprised of the indicators variables: PACS, COUNT, CIWA, DRINC, and ADS, all of which are described previously (and was tested both with and without the count indicator variable); and (b) test the relationship between the AUD severity factor and measures of alcohol use, affective symptoms, and motivation to change drinking behavior. Further, it was hypothesized that there would be significant inter-factor correlations between the three validation constructs (i.e., alcohol use, affective symptoms, and motivation for change), hence inter-factor covariances were estimated between these domains. The latent constructs included observed variables as described in the measures section (AUD Severity, Alcohol Use, Affective Symptoms, and Motivation to Change). Variances for independent latent constructs were fixed to one with all indicator paths freely estimated. The variance for the AUD severity construct was freely estimated with the ADS path coefficient set to one. All errors were freely estimated. Maximum likelihood modeling analyses were conducted on the raw data using the EQS version 6.1 for Windows SEM program (22). Robust statistical estimates are reported due to the non-normal distribution of the alcohol indicator variables. Statistical model fit was assessed with the Satorra–Bentler scaled chi-squared fit index (23). A relative estimate (ratio of chi-square to degrees of freedom) was also calculated, as the use of the chi-squared likelihood ratio to assess the model fit has been deemed unsatisfactory for numerous reasons (24). Values<2 on the relative chi-square indicate adequate model fit (25). Descriptive model fit was assessed with the robust versions of the comparative fit index [CFI; (26)] and the root mean square error of approximation [RMSEA; (27)]. Both the CFI and the RMSEA are sensitive to model misspecifications and are minimally affected by sample size (28). The CFI ranges from 0 to 1, with values above 0.90 indicating acceptable fit (26). The RMSEA ranges from 0 to 8, where fit values<0.05 indicate close fit and values<0.10 indicate reasonable fit (29).

## Results

### Descriptive statistics

A total of 295 participants completed the in-person assessment battery. Six subjects were removed from the analyses for psychiatric disorders as determined by the SCID, and 11 were excluded because of missing data on one or more of the measures, leaving a total of 283 subjects (75 women, 205 men). Of these 283 subjects, 71.86% met DSM-IV criteria for alcohol dependence, 11.83% met criteria for alcohol abuse only, 12.19% were diagnostic orphans, and 4.3% did not endorse any symptoms of either alcohol abuse or dependence. Means, standard deviations, and correlations across all model variables are presented in Table 1. Depression and anxiety disorders were not evaluated using a diagnostic interview; however symptomatology was self-reported via the BDI-II and BAI measures. Clinical cut-off scores were assessed to determine severity of mood and affective symptoms (30, 31). On the BDI-II, 33% reported in the minimal range (0–13), 10% scored in the mild range (14–19), 32% scored in the moderate range (20–28), and 25% reported in the severe range (29–63). For self-reported anxiety, 29% reported in the minimal range (0–7), 15% scored in the mild range (8–15), 24% reported moderate symptoms (16–25), and 31% scored in the severe range (26–63).

**Table 1:** **Means, standard deviation (SD), and correlations for all observed model parameters**.

	Mean	SD	1	2	3	4	5	6	7	8	9	10	11	12
1. ADS	40.25	7.31	1											
2. PACS	17.92	6.61	0.50^*^	1										
3. COUNT	5.23	2.81	0.48^*^	0.35^*^	1									
4. DRINC	40.9	22.12	0.65^*^	0.52^*^	0.54^*^	1								
5. CIWA	5.66	6.92	0.23^*^	0.27^*^	0.30^*^	0.23^*^	1							
6. STEPS	2.8	0.93	0.24^*^	0.16^*^	0.28^*^	0.35^*^	0.09	1						
7. RECOG	2.72	0.92	0.48^*^	0.48^*^	0.51^*^	0.68^*^	0.21^*^	0.57^*^	1					
8. AMBIV	3.09	0.92	0.44^*^	0.44^*^	0.46^*^	0.58^*^	0.15^*^	0.61^*^	0.80^*^	1				
9. BAI	18.82	12.99	0.29^*^	0.29^*^	0.04	0.28^*^	0.05	0.07	0.23^*^	0.13^*^	1			
10. BDI	20.56	12.04	0.23^*^	0.32^*^	0.06	0.27^*^	0.07	0.1	0.24^*^	0.17^*^	0.83^*^	1		
11. DPDD	7.08	4.66	0.26^*^	0.26^*^	0.22^*^	0.20^*^	0.22^*^	0.05	0.20^*^	0.17^*^	0.03	0.002	1	
12. BINGE%	0.66	0.3	0.33^*^	0.22^*^	0.36^*^	0.33^*^	0.25^*^	0.11	0.31^*^	0.28^*^	0.02	−0.004	0.62	1

*p < 0.05. ADS, alcohol dependence scale; PACS, alcohol craving score; COUNT, alcohol dependence and abuse symptom count; DRINC, negative drinking-related consequences; CIWA, alcohol withdrawal score; STEPS, taking action to change drinking; RECOG, recognition of alcohol-related problems; AMBIV, uncertainty about drinking; BAI, anxiety symptoms; BDI, depressive symptoms; DPDD, drinks per drinking days; BINGE%, percent binge drinking days.

### SEM model results

**Figure 1:**
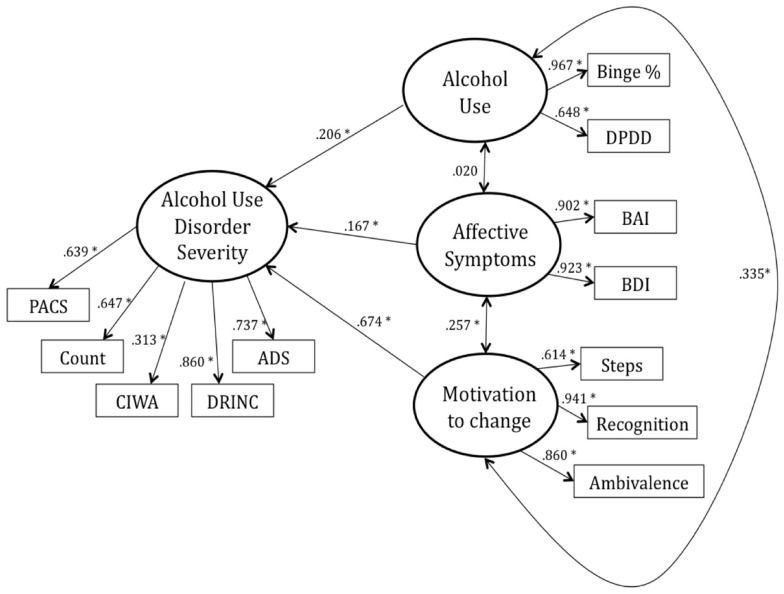
**The final estimated model with standardized path coefficients (ß)**. The following results are based on the final model with the standard significance threshold at*p* < 0.05.

The model was found to fit well descriptively (CFI = 0.960, RMSEA = 0.064) and relatively well statistically [S–B scaled χ^2^ (48, *n* = 283) = 103.73; relative χ^2^ = 2.16]. The final estimated model, with standardized path coefficients, is presented in Figure 1. The Alcohol Use, Motivation, and Affective Symptoms paths accounted for 68% of the variance in AUD severity. The following results are based on the final model with the standard significance threshold at *p* < 0.05.

Importantly, the AUD severity indicator variables, COUNT (β = 0.647), CIWA (β = 0.313), PACS (β = 0.639), DRINC (β = 0.860), and ADS (β = 0.737), were all found to load significantly onto the AUD severity latent construct, suggesting that all indicators are explaining variance from the same construct. The affect symptomatology indicator variables, BDI (β = 0.923) and BAI (β = 0.902), loaded significantly onto the Affective Symptoms factor. Additionally, DPDD (β = 0.648) and percent binge drinking days (Binge%; β = 0.967) loaded significantly onto the Alcohol Use factor and the three indicator variables on the Motivation factor loaded significantly (Steps, β = 0.614; Recognition, β = 0.941; Ambivalence, β = 0.860).

As expected, the path from Alcohol Use to AUD severity was found to be statistically significant (β = 0.206), such that greater AUD severity was associated with higher levels of alcohol use. The path from Affective Symptoms to AUD severity was found to be significant (β = 0.167) as well as the path from Motivation to AUD severity (β = 0.674). The relationships were such that greater AUD severity predicted increased anxiety/depression symptoms as well as greater motivation for change. There was a statistically significant and positive inter-factor correlation between Affective Symptoms and Motivation (*r* = 0.257) and between Alcohol Use and Motivation (*r* = 0.335), such that individuals reporting greater anxiety/depressive symptoms as well as those who drank more heavily endorsed higher motivation to change their drinking. However, the relationship between Alcohol Use and Affective Symptoms was not found to be statistically significant (*r* = 0.020).

To investigate the influence of the 12 subjects who did not endorse any AUD symptoms as assessed by the SCID, the model was re-estimated with these subjects removed from the analysis and was found to be virtually identical to the original model [CFI = 0.956, RMSEA = 0.067; S–B scaled χ^2^ (48, *n* = 271) = 105.40; relative χ^2^ = 2.20]. Further, to investigate the possibility of gender differences, a group analysis on the original model was conducted by constraining all paths in the model to be equal across gender. The Lagrange multiplier test, used to test the gender hypothesis in a maximum likelihood framework whereby the hypothesis being tested is expressed as one or more constraints on the values of parameters (32), revealed two constrained paths to be released. These include the paths between BAI and Affective Symptoms (χ^2^ = 8.26, *p* < 0.05) as well as between BDI and Affective Symptoms (χ^2^ = 5.07, *p* < 0.05), suggesting that influence of the BAI and BDI on the Affective Symptoms factor in the context of this model differ between genders.

The second model which excluded the symptom count indicator variable (COUNT) from the AUD severity factor resulted in estimates that were largely equivalent to the original model, such that the remaining alcohol severity indices loaded significantly onto the severity factor. The second model was also found to fit well descriptively (CFI = 0.969, RMSEA = 0.064) and relatively well statistically [S–B scaled χ^2^ (35, *n* = 283) = 76.13; relative χ^2^ = 2.17]. This suggests that a meaningful AUD severity factor may be obtained in the absence of diagnostic data (i.e., symptom count), which is relevant in the context of practicality in a clinical setting.

## Discussion

This study sought to evaluate and validate an AUD severity factor by assessing the influence of various indices of AUD and testing the factor’s relationship to alcohol use, motivation for change, and affective symptoms using a SEM approach. The AUD severity factor is comprised of quantitative scores from self-report and interview assessments that intentionally measure the multiple dimensions and variability of impairment observed across the progression of the disorder. Additionally, the AUD severity factor combines all 11 symptoms of AUD into a single variable (COUNT) which eliminates the “diagnostic orphan” category. Although each indicator variable captured different aspects of AUD, all the AUD severity indicator variables loaded significantly onto a single factor, and together, greater AUD severity was associated with greater alcohol use, increased affective symptoms, and higher motivation to change; thus validating the construct’s utility in clinical settings and in research.

Neurobiological theory points to the importance of assessing the progression of AUD as the various stages of severity are associated with different outcomes. For example, the persistence of the psychomotor sensitization (i.e., incentive salience) observed in severely dependent preclinical samples (5) could help explain why some individuals with alcohol dependence are more susceptible to cue-induced relapse than others. In addition, the neurobiological theories supported by neuroimaging studies of addiction suggest that treatment response may depend, at least in part, on the severity of addiction. As an example, if greater alcohol dependence severity is in fact accompanied by the shift to more striatal driven habitual behavior, treatments targeting cognitive control over one’s drug use may prove futile.

Currently, evidence-based treatments for alcohol disorders largely rely on the DSM-IV categorical clinical diagnosis of abuse and dependence. However, the field is moving toward a broader understanding of alcohol disorders as reflected by the ongoing efforts to change the categorical DSM system to a dimensional system (33) where individuals diagnosed with an AUD will be rated on a severity scale ranging from mild to severe (dsm5.org). The decision to include a singular AUD diagnosis in DSM-5 represents a shift from the previous distinction between Alcohol Abuse and Dependence in DSM-IV. This change is founded on an overall goal of simplifying the diagnostic process for clinicians and a sizeable amount of research suggesting that substance use disorders are more adequately represented by a continuum of severity (34). Further, under this new system, those deemed diagnostic orphans based on DSM-IV criteria may meet for a mild AUD if two symptoms are endorsed. Interestingly, however, removal of the DSM-IV count variable from the AUD severity factor did not alter the relationships in the present study model or the overall model fit. This finding implies that the categorical assessment of DSM-IV alcohol abuse and dependence symptoms (together roughly equating to the DSM-5 criteria of AUD) provides little information beyond what is measured by the self-report indices. This is relevant for clinical practice, as the assessment of DSM-IV, and soon to be DSM-5, symptomatology requires a lengthy diagnostic interview that must be administered by trained staff. Thus, it is promising that quantitative indices of severity can be derived from self-report data and in the absence of diagnostic data.

The variability in AUDs phenomenology has challenged researchers and clinicians to generate personalized treatments that take into account specific clinical profiles (8). These efforts have been successful in studying response to medications for AUD based on individuals’ genetic make-up (35, 36). As the field awaits improvements to make available genetic testing to guide treatment, having instruments that facilitate the evaluation of AUD severity offer several advantages that may improve the efficacy of evidence-based treatments. Administering and scoring these measures may prove cost effective as clinicians may use the information on AUD severity to determine whether patients should undergo a detoxification program, would benefit from inpatient treatment, or are able to comply with outpatient programs. Similarly, information on AUD severity may also guide clinician’s judgment regarding recommendations for medication for AUDs as studies have shown that certain treatment work better for more or less severe patients [e.g., quetiapine for high severity, very heavy drinkers; (37)]. In brief, information on AUD severity can improve treatment efficacy by guiding clinician’s decision processes based on empirical data.

Of note, alcohol use *per se*, was not included as a indicator variable of the severity factor as the goal of this paper was to validate and extend the AUD severity factor previously developed by our research team (12, 38). We considered testing alcohol use as an indicator but decided against it as the factor loadings of the clinical scales selected are much higher than the correlation between alcohol use and the AUD severity factor.

This study must be interpreted in light of its strengths and weaknesses. This was a cross-sectional examination of problem drinkers, thereby precluding causal inferences about the progression of the disorder within individual subjects and about the relationships between AUD severity and outcome variables. The exclusion of treatment-seeking individuals, those who met criteria for a lifetime DSM-IV diagnosis of psychiatric disorders, and current use of psychoactive drugs, limits the generalizability of the findings. However, as this was part of a larger-scale alcohol administration study (12) the exclusionary criteria were designed with safety and ethical concerns in mind. Additionally, although considered large for the nature of the sample, the sample size is not sufficient for further in-depth inquiry into sample characteristics such as the influence of ethnicity within the specified SEM model. Future studies might address these limitations by recruiting larger samples within a longitudinal framework.

In summary, this study validated an AUD severity factor in relation to alcohol use, mood symptomatology, and motivation to change drinking behavior in a well-characterized community sample of individuals with AUD. Ultimately, the goal is for the AUD severity factor to be tested in biologically based models, such as in neuroimaging (38, 39) and to be utilized by clinicians in order to provide services that more effectively target patient’s needs. The successful development and validation of an AUD severity scale can be used to develop tailored treatment plans for alcohol dependent patients at various stages of the disorder. Likewise, capturing AUD severity has broad implications for research, as it will facilitate the application of clinical neuroscience approaches to AUDs.

## Conflict of Interest Statement

Lara A. Ray is a paid consultant to GlaxoSmithKline. All other authors report no conflicts of interest. The authors alone are responsible for the content and writing of this paper.
